# Role of Gut Microbiota on Onset and Progression of Microvascular Complications of Type 2 Diabetes (T2DM)

**DOI:** 10.3390/nu12123719

**Published:** 2020-12-02

**Authors:** Daniela Maria Tanase, Evelina Maria Gosav, Ecaterina Neculae, Claudia Florida Costea, Manuela Ciocoiu, Loredana Liliana Hurjui, Claudia Cristina Tarniceriu, Minela Aida Maranduca, Cristina Mihaela Lacatusu, Mariana Floria, Ionela Lacramioara Serban

**Affiliations:** 1 Department of Internal Medicine, “Grigore T. Popa” University of Medicine and Pharmacy, 700111 Iasi, Romania; tanasedm@gmail.com (D.M.T.); floria.mariana@umfiasi.ro (M.F.); 2 Internal Medicine Clinic, “St. Spiridon” County Clinical Emergency Hospital Iasi, 700115 Iasi, Romania; 3 Department of Gastroenterology, “Grigore T. Popa” University of Medicine and Pharmacy, 700115 Iasi, Romania; ecaterina.neculae91@gmail.com; 4 Institute of Gastroenterology and Hepatology, “St. Spiridon” County Clinical Emergency Hospital Iasi, 700111 Iasi, Romania; 5 Department of Ophthalmology, “Grigore T. Popa” University of Medicine and Pharmacy, 700115 Iasi, Romania; claudia.costea@umfiasi.ro; 6 2nd Ophthalmology Clinic, “Nicolae Oblu” Emergency Clinical Hospital, 700309 Iași, Romania; 7 Department of Pathophysiology, Faculty of Medicine, “Grigore T. Popa” University of Medicine and Pharmacy, 700115 Iasi, Romania; manuela.ciocoiu@umfiasi.ro; 8 Department of Morpho-Functional Sciences II, Physiology Discipline, “Grigore T. Popa” University of Medicine and Pharmacy, 700115 Iasi, Romania; loredana.hurjui@umfiasi.ro (L.L.H.); minela.maranduca@umfiasi.ro (M.A.M.); ionela.serban@umfiasi.ro (I.L.S.); 9 Hematology Laboratory, “St. Spiridon” County Clinical Emergency Hospital, 700111 Iasi, Romania; 10 Department of Morpho-Functional Sciences I, Discipline of Anatomy, “Grigore T. Popa” University of Medicine and Pharmacy, 700115 Iasi, Romania; claudia.tarniceriu@umfiasi.ro; 11 Hematology Clinic, “St. Spiridon” County Clinical Emergency Hospital, 700111 Iasi, Romania; 12 Unit of Diabetes, Nutrition and Metabolic Diseases, “Grigore T. Popa” University of Medicine and Pharmacy, 700115 Iasi, Romania; cristina.lacatusu@umfiasi.ro; 13 Clinical Center of Diabetes, Nutrition and Metabolic Diseases, “St. Spiridon” County Clinical Emergency Hospital, 700111 Iasi, Romania; 14 Internal Medicine Clinic, Emergency Military Clinical Hospital, 700483 Iasi, Romania

**Keywords:** gut microbiota, dysbiosis, type 2 diabetes, retinopathy, neuropathy, nephropathy, therapy

## Abstract

Type 2 diabetes mellitus (T2DM) remains one of the most problematic and economic consumer disorders worldwide, with growing prevalence and incidence. Over the last years, substantial research has highlighted the intricate relationship among gut microbiota, dysbiosis and metabolic syndromes development. Changes in the gut microbiome composition lead to an imbalanced gastrointestinal habitat which promotes abnormal production of metabolites, inflammatory status, glucose metabolism alteration and even insulin resistance (IR). Short-chain fatty acids (SCFAs), trimethylamine N-oxide (TMAO), lipopolysaccharide, aromatic amino acids and their affiliated metabolites, contribute to T2DM via different metabolic and immunologic pathways. In this narrative review, we discuss the immunopathogenic mechanism behind gut dysbiosis, T2DM development and the major known diabetic microvascular complications (retinopathy, neuropathy and nephropathy), the beneficial use of pre- and pro-biotics and fecal microbiota transplantation in T2DM management and new findings and future perspectives in this field.

## 1. Introduction

Type 2 diabetes mellitus (T2DM) remains one of the most problematic chronic metabolic disorders, affecting about 422 million people worldwide with steadily increased incidence and prevalence over the past decade, 90% of whom have type 2 diabetes mellitus (T2DM). According to World Health Organization (WHO) and International Diabetes Federation (IDF), about 629 million people will have T2DM by 2045; a further 352 million people with impaired glucose tolerance are at great risk for diabetes debut; and there is a globally agreed target to halt the rise in diabetes and obesity by 2025 [1,2]. Diabetes is a major cause of blindness, kidney failure, heart attacks, stroke and lower limb amputation and is clinically hallmarked by hyperglycemia, insulin resistance (IR) and pancreatic β-cell decompensation [3].

Obesity-induced IR is one of the main triggers of T2DM development and evidence shows that IR is also governed by the gut microflora. Over that last years, abundant research has tried to delve into the profound gut microbiota (GM) matrix and find its exact relationship with diabetes and its complications. The GM atlas is complex and various, and it has a crucial role in the homeostasis and function of other organs and diseases. GM dysbiosis can extend its effects beyond the gastrointestinal tract, including chronic illnesses such as diabetes and its major and minor vascular complications [4]. Many gut compositional changes have been reported in obese and T2DM patients, compared to healthy subjects, and gut microflora may be a significant environmental factor involved in the onset and progression of diabetes. Moreover, intestinal dysbiosis was also associated with type 1 diabetes mellitus and gestational diabetes mellitus onset [5,6]. Because of treatment inertia, substantial efforts are currently made into exploring the thick substrate of T2DM pathophysiology and find new therapeutic targets. Thus far, the known documentation in this field hints at the significant role of the GM alterations in the onset and evolution of the microvascular complications of diabetes [7].

In this review, we discuss the immunopathogenic mechanism behind gut dysbiosis-TD2M development, the major known diabetic microvascular complications (retinopathy, neuropathy and nephropathy), the current knowledge on the beneficial use of pre- and pro-biotics and fecal microbiota transplantation (FMT) in diabetes management and the new recently findings in this field.

## 2. Materials and Methods

We conducted an extensive research using literature published over the last five years, up until August 2020, regarding the intricate mechanisms that connect GM dysbiosis with the development of T2DM and its microvascular complications. The US National Library of Medicine (PubMed), Scopus and Google Scholar were the electronic databases used as sources for relevant articles related to our subject. Studies published between the 1 January 2015 and the 1 August 2020 were selected to avoid any outdated data. We used different combinations of the following keywords: “gut microbiota”, “dysbiosis”, “type 2 diabetes”, “immunologic pathways”, “retinopathy”, “neuropathy”, “nephropathy” and “therapy”. Various article types such as clinical trials, randomized controlled trials, multicenter studies, reviews, guidelines and meta-analysis were included. Only publications that were available in full text and in English were of interest. In addition, eligible and relevant articles were obtained from the reference lists of the previously selected articles. Two reviewers (E.M.G. and E.N.) primarily screened the articles by title and abstract. After that, under the supervision of a third reviewer (D.M.T.), they proceeded to full-text evaluation. The focus of this narrative review is on three major key-points. All the relevant information extracted from the selected articles is summarized in text form. Firstly, we summarize the newly found connection between the gut microbiota compositional changes and the development of metabolic disorders such as T2DM, especially the immunological pathway induced by products of gut microbial metabolites. After that, we show how these pathological changes lead to microvascular complications (retinopathy, neuropathy and nephropathy) associated with T2DM. The last part of this review is focused on potential therapeutic options.

## 3. Gut Microbiota and T2DM

In the last years, studies on gut biodiversity content and function in subjects with metabolic disorders have considerably increased, particularly on type 1 diabetes and T2DM complications. Dysbiosis and/or leaky gut syndrome encompasses an imbalanced distribution of bacteria population and impairment of bacterial metabolic activity which increases the intestinal permeability with subsequently multiple dysregulations [7,8]. The human body is habituated by almost one hundred trillion microbes that belong to 2000 species, classified into 12 different phyla that mainly reside in the gastrointestinal tract, which accounts for 80% of the total microbiota. The microbiome is also characterized by microorganisms and their genomes, which display about 150 times more genes than the human genome [8]. The various content of the GM is represented, besides bacteria, by viruses (mainly phages), archaea, protozoa and, eukaryotes (mainly yeasts and fungi), although they are found in a much smaller concentration. Five main phyla embody the GM: *Actinobacteria*, *Bacteroidetes*, *Firmicutes*, *Proteobacteria* and *Verrucomicrobia* [9]. The phyla species constituted mostly of *Firmicutes* (Gram-positive) and *Bacteroidetes* (Gram-negative) account for 90% of the total population, and the rest of *Actinobacteria* and *Proteobacteria*, which promote the uptake of monosaccharides, enhancing the hepatic triglyceride production with subsequently IR [10].

The GM is a very diversified ecosystem, and its architecture and function is adjusted through life and is dependent on several factors such as host genetics, species, gender, age, weight, height, demographics, environment, diet and socioeconomic factors, as well as smoking, antibiotics and antibiotics-like substances, laxatives and drugs such as antihistamines, antidepressants and metformin [11,12]. Food intake and body weight can be modulated by species such as *Clostridium hystoliticum*, *Clostridium coccoides* and *Eubacterium rectale*, which can disturb neurotransmitters that directly control the transit time, permeability and physical activity of the intestinal wall [13]. GM biodiversity contributes to essential vitamin production (e.g., vitamin K and B12), biosynthesis of glycans, amino acids, methanogenesis isoprenoids, development of regulatory T-cells and metabolization of many products including xenobiotics or toxins [14,15,16].

A variety of research has brought attention about the function of the gastrointestinal microbe population in modulating host health and physiology, with roles in regulating the impairment of other distant organs/systems as a reaction to different injuries such as cardiovascular [17], neurological [18], hepatic [19] or renal injury [20]. Among the first reports regarding the gut dysbiosis-diabetes relationship, Larsent et al. [21] found that the *Bacteroidetes:Firmicutes* ratio and *Bacteroides–Prevotella* group to *Clostridium coccoides–Eubacterium rectale* group positively linked with serum glucose levels, and that diabetic subjects have decreased butyrate-producing bacteria levels (*Clostridiales* spp., *Faecalibacterium prausnitzii*, *Eubacterium rectale*, *Roseburia* intestinalis and *R. inulinivorans*) and increased abundance of *Lactobacillus* species. Proteobacteria incorporate many pathobionts, which may have a pro-inflammatory effect in diabetic subjects through their specific lipopolysaccharides. Pyrosequencing of the V4V5 region of 16S rRNA genes revealed a notable decrease of butyrate-producing bacteria such as *Akkermansia* and *Bifidobacterium* with an abundance of *Dorea* spp. in the diabetic patient [22], and a Mendelian randomization study found that *Acidaminococcus*, *Anaerostipes*, *Aggregatibacter, Blautia*, *Dorea*, *Desulfovibrio* and *Faecalibacterium* gut concentrations are also associated with diabetes [23]. Another study noted that the genera of *Bifidobacterium*, *Bacteroides*, *Faecalibacterium*, *Akkermansia* and *Roseburia* were negatively associated with T2DM, while the genera of *Ruminococcus*, *Fusobacterium* and *Blautia* were positively associated with diabetes. *Fusobacterium* is a phylum that adds adhesiveness to host epithelial cells and contributes to inflammatory responses [24]. Some suggest that decreased *Akkermansia muciniphila* could be used as a biomarker for the early diagnosis of diabetes [25]. In addition, researchers noted that T2DM patients had lowered levels of *Bifidobacterium* spp., compared to control patients [26], and that the relative abundance of *Firmicutes* and *Actinobacteria* was positively correlated with fasting glucose levels, while *Bacteroidetes* and *Proteobacteria* exhibited a negative correlation [27]. *Bacteroides* and other commensal bacteria can affect the intestinal permeability because they can alter the intestinal mucus and glycocalyx. This process was linked with a high-fat diet. Meanwhile, *Bifidobacterium* spp. has anti-inflammatory properties and protects the tight junctions in the epithelial barrier [28].

A cross-sectional study presented that healthier dietary-prediabetic subjects have a decrease in *Prevotella* and an increase of *Faecalibacterium prausnitzii*. There was also an increase in lactic acid bacteria. These results suggest that the growth of beneficial bacteria in patients with healthy diets could protect against the development of T2DM in an obese population with advanced age and prediabetes [29]. Functional correlation analysis noted that modified *Verrucomicrobiaceae* and *Bacteroidaceae* gut levels significantly correlated with alterations in fecal metabolites [30], and obese-T2DM patients have an absence of *Verrucomicrobia* phyla, a microorganism that has anti-inflammatory gut effects and helps improve insulin sensitivity [31]. T2DM Japanese subjects had diminished fecal butyrate and propionate levels, higher fecal of *Bifidobacterium* spp. and bacteria of the order *Lactobacillales* and lower fecal *Bacteroides* spp. populations than the control subjects. In addition, the level of *Lactobacillales* species correlated negatively with the protein intake, while the level of *Bifidobacterium* spp. correlated negatively with the carbohydrate intake [32]. Others found that T2DM-obese subjects have lower levels of *L. acidophilus*, *L. plantarum* and *L. reuteri* subgroups of *Lactobacillus* spp. compared to controls [33].

As seen, diabetic patients have increased gram-negative bacteria such as *Bacteroides* which can enhance succinate, propionate and acetate production from lactate, which then may damage the epithelial cell barrier permitting pathogen to enter the cell. Further, various studies pointed out that auxiliary microbial functions were enhanced during dysbiosis resulting in oxidative stress resistance which further delineates the beneficial use of GM alteration as possible predictive biomarkers for diabetes. The GM is involved in the development of different organ damage, hence targeting the gut microflora could represent a future therapeutic approach in diseases such as T2DM and its complications.

## 4. The Imunopathogenesis behind Gut Dysbiosis-T2DM

A plethora of evidence highlights the pivotal role of GM dysbiosis in obesity and T2DM pathogenesis. T2DM includes β-cell dysfunction, derangement of lipid and glucose metabolism, chronic low-grade inflammation and oxidative stress, which result in IR and insufficient insulin secretion [1,2]. Energy disbalance, nonspecific humoral, gut dysbiosis and other cellular responses accompany the chronic inflammation of diabetes. A wide spectrum of scientific research has shown how GM influences the host through different pathways including products of gut microbial metabolites. Microbial metabolites are key mediators of microbial–host crosstalk, impacting the organism’s glucose metabolism. The intestinal microbiota produces a few metabolites such as short-chain fatty acids (SCFAs), amino acids, trimethylamine N-oxide (TMAO), bile acids and indole propionic acids, which participate in the regulation of host metabolism and gut integrity [34,35]. Via these products, GM moderates and imprints its complex effects on diseases such as diabetes.

### 4.1. Short-Chain Fatty Acids (SCFAs)

SCFAs are one of the primary end products of bacteria fermentation that have considerable effects on host physiology and serve as signaling molecules between intestinal microbiota and the host. The complex consumed carbohydrates are metabolized by colonic bacteria into monosaccharides and oligosaccharides, and they are fermented into SCFAs and gases (hydrogen, methane and carbon dioxide). SCFAs endorse the production and release of cytokines, chemokines, protective peptides and phagocytes. They regulate lipid and glucose metabolism by activating the SCFAs hepatic, adipose tissue, pancreas and brain cell receptors. In addition, SCFAs represent the main source of energy for enterocytes and colonocytes and are able to inhibit intestinal inflammation and oxidative stress [36]. The residential microbiota in the cecum and colon metabolizes the dietary fibers, peptides and proteins that escaped the upper gut digestion. At this intestinal level, they help biosynthesis of the three major SCFAs (acetic acid, propionic acid and butyric acid) through glycolysis of glucose to pyruvate and acetyl-CoA, after which these products are derived. The resulted SCFAs are usually used as material for the colonic mucosal epithelial cells or go into the portal circulation impacting the host’s well-being. They stimulate glucagonlike peptide-1 (GLP-1) and GLP-2 secretion via a G-protein-coupled receptor (GPR), with increased expression of adiponectin and insulin and subsequent amplification of insulin sensitivity and pancreatic s-cells proliferation. Interestingly, both glucose homeostasis and satiety are mediated by GM that enhances GLP-1 secretion [37,38].

A hallmark of T2DM is IR, usually associated with obesity, metabolic syndrome and intestinal composition alterations with higher levels of lipopolysaccharide (LPS)-enriched Gram-negative bacteria which favor increased cellular permeability. There is a paucity of the bacterial population that can engender beneficial SCFAs, which stabilizes macrophages against inflammation and can shelter the gut endothelial barrier [39]. Metabolic endotoxemia is triggered by gut dysbiosis and mirrors a low-grade systemic inflammation in response to LPS and peptidoglycan (PG) uptake by the systemic bloodstream, in high-fat diet conditions. This process enhances activation of toll-like receptor-4 (TLR-4) and other inflammatory signaling pathways, which further promote IR and T2DM [40]. The SCFAs that derive from fermentation bind to GPR41 and GPR43 to create enteroendocrine molecules that mediate appetite and the intestinal gluconeogenesis gene expression. Interestingly, the pancreatic β-cells express these receptors that participate in insulin secretion. For example, GPR41 knockout rodents have altered glucose control through SCFA-activated GPR41 signaling that regulates pancreatic insulin secretion [41]. These findings highlight the key role of SCFAs-GPR signaling in serum glucose homeostasis and insulin metabolism.

Zhao et al. [42] demonstrated that a GM that is selectively sustained by high-dietary fibers led to increased butyrate- and acetate-producing microbiome products, improved glucose intolerance, lowered hemoglobin A1c levels and overall alleviated T2DM. Notably, acid acetic embodies more than half of the SCFAs and the dominant acetate-producing bacteria are represented by *Clostridium* spp., *Streptococcus* spp., *Prevotella* spp. and *Bifidobacterium* spp. [43]. While the major propionate-producing bacteria are represented by *Bacteroides* spp., *Salmonella* spp., *Dialister* spp., *Coprococcus Catus*, *Blautia obeum* and many others, the main butyric acid-producing bacteria are represented by *Ruminococcaceae*, *Lachnospiraceae* and *Acidaminococcaceae* families. Butyrate is one of the main energy sources of the gut epithelium and increased levels can induce gut gluconeogenesis which reduces the production of liver glucose, decreases body weight and improves the intestinal wall function. Using bidirectional Mendelian randomization (MR) analyses, the authors found that the host-genetic-driven increase in gut production of the SCFA butyrate was linked to better insulin response after an oral glucose tolerance test, while flawed production or absorption of propionate was correlated to a raised risk of diabetes [44]. On the other hand, propionate protects against diet-induced obesity and glucose intolerance by being one of the substrates for gluconeogenesis [45]. In vitro and in vivo studies showed that propionate sustained β-cell mass by suppressing cell apoptosis, and that colon insertion of inulin-propionate ester improved β-cell function [46].

### 4.2. Bile Acids (BAs)

Cumulative data gathered especially in the last years describe the role of bile acids (BAs) in diabetic patients and diabetic animals [47,48]. Usually, bile acids are synthesized from cholesterol in the hepatic parenchyma under the two forms chenodeoxycholic acid (CDCA) and cholic acid (CA) via a classical pathway, while the alternative pathway generates mainly CDCA [49]. The intestinal microbiota then dihydroxylates, deconjugates, dehydrogenates and proceeds to the epimerization of the primary BAs into secondary BAs, mainly by the action of *Firmicutes*. The secondary BAs are expressed as deoxycholic acid (DCA), hyodeoxycholic acid (HDCA), lithocholic acid (LCA), x-muricholic acid (xMCA), hyocholic acid (HCA) and murideoxycholic acid (MDCA). They are stored in the gallbladder. When necessary, the secondary BAs are released into the duodenum to help digestion. After they complete their job, these acids are then reabsorbed in the ileum and transferred via the portal circulation to the liver. The primary BAs activate the nuclear farnesoid X receptors (FXR) which alter the glucose metabolism, while the secondary ones bind to the G protein-coupled bile acid receptor 1 (GPBAR 1/TGR5) that promotes glucose homeostasis [49]. Individuals with uncontrolled T2DM have raised BAs with increased deoxycholic acid (DCA) and less CDCA levels [50].

Because of their ability to mediate energy metabolism by binding and activating nuclear transcription factors such as FXR in the intestine and liver, manipulation of BAs via adjustment of the GM could help glycemic control and prevent metabolic memory for early-onset T2DM subjects [51]. BAs sequestrants have been recently introduced as therapeutic options in patients with inadequately controlled diabetes by normal antidiabetic treatment. A recent molecule approved by the Food and Drug Administration (FDA) is colesevelam. It can reduce low-density lipoprotein (LDL) cholesterol and total cholesterol, whole-body insulin sensitivity and insulin release; four phase III clinical trials provided evidence that colesevelam, as a monotherapy and/or added to other glucose-lowering treatments confers placebo-corrected reductions in HbA1c of ~5 mmol/mol [52]. In addition, administration of the semisynthetic BA derivative obeticholic acid (OCA), a high-affinity ligand of FXR, showed controversial results in diabetic subjects [53]. Using the TGR5 agonists and FXR agonists via GM modulation may serve as a potential therapeutic approach for the treatment of T2DM.

### 4.3. Trimethylamine Oxide (TMAO)

Among the resulted fermented products by the intestinal biodiverse bacteria are amines and polyamines. The intestinal bacteria help the formation of metabolized trimethylamine (TMA) from choline, which is then transported to the liver, oxidized by flavin monooxygenase 3 (FMO3) and transformed into trimethylamine oxide (TMAO). TMAO has pro-atherogenic properties. The original compounds that with the help of gut bacteria are transformed into TMA via hepatic FMO are represented by choline, phosphatidylcholine, carnitine, c-butyrobetaine, betaine, crotonobetaine and glycerophosphocholine. Choline is a salient nutrient needed for lipid metabolism and hepatic production of very-low-density lipoproteins (VLDLs) [54]. TMAO has an important role in the onset and maintenance of T2DM and is correlated with a significant risk of other metabolic syndromes [55]. Studies describe different mechanisms by which TMAO influences glucose metabolism. Gender, age and common family habits, such as diet (high red meat and fish intake), contribute to variation in serum concentrations of TMAO and its precursors [56]. In vivo studies show that bacteria such as *Firmicutes*, *Proteobacteria*, *Clostridium asparagiforme*, *C. hathewayi*, *C. sporogenes*, *Escherichia fergusonii*, *Proteus penneri*, *Providencia rettgeri*, *Edwardsiella tarda* and *Desulfovibrio desulfuricans* are TMAO-generators [57].

The whole landscape that incorporates the TMAO metabolism, its association with microbial dysbiosis and correlation with cardiovascular, neurological, renal disease and cancer proposes TMAO as a potent novel biomarker [58]. The POUNDS Lost trial shows that overweight and obese adults who were assigned to energy-reduced diets varying in macronutrient intake, over six months, noted greater decreases in choline and L-carnitine levels associated with greater improvements in fasting insulin concentrations and homeostasis model assessment of insulin resistance (HOMA-IR) [59]. TMAO is a well-known pro-atherogenic agent and evidence suggests that GH composition changes are accompanied by arterial stiffness and endothelial dysfunction. This may explain the implications of GM dysbiosis in the microvascular injury of diabetes, even if there is no conclusively proven research regarding this association [60].

### 4.4. Indole Propionic Acids and Branched Chain Amino Acids (BCAAs)

Around 10 g of proteins are degraded by GM every day to metabolites such as amines, thiols, phenols, indoles and ammonium. Indole-3-propionic acid or 3-Indolepropionic acid (IPA) is generated by the intestinal microbiota endogenously using tryptophan, absorbed by the gut epithelium and then transferred into circulation. Other relevant results from tryptophan degradation are indoxyl sulfate and indoleacetic acid [61]. IPA may improve glucose metabolism and has antioxidant and anti-inflammatory effects. Plasma IPA may be a potential biomarker for diabetes as it is linked to T2DM development and it can exert protective effects by maintaining the β-cell function [62]. Interestingly, GM can generate or modify xenometabolites. Developing a metabolomics platform (“XenoScan”, University of California, Davis, USA) using liquid chromatography–mass spectrometry to describe xenometabolites, the author showed that, in T2DM rodents, some xenometabolites such as IPA negatively correlated with *Oscillibacter* spp., whereas gluconic acid and TMA positively correlated with *Oscillibacter* species. This new assay may enable rapid identification of a wide variety of gut-derived metabolites and discover new insights in the gut microbial ecology and xenometabolite relationship [63].

Branched-chain amino acids (BCAAs) are key components in glucose and protein metabolism. Dysbiosis can alter the decomposition of BCAA and promote oxidative stress responses and embellishment in membrane cell transportation of BCAA and sugars [64]. BCAAs are produced mainly by *Prevotellacopri* and *Bacteroides vulgatus* spp. [65]. Raised serum levels of BCAAs may predict the development of T2DM. Data show that short-term dietary reduction of BCAAs improves the metabolism of white adipose tissue and intestinal microbiota composition and lowers the postprandial insulin secretion [66].

### 4.5. Hydrogen Sulfide (H_2_S)

The gut composition assists Hydrogen Sulfide (H_2_S) production from fermented proteins. Recently it was brought into attention the impact of H_2_S and dysbiosis on the organism signaling and function [67]. H_2_S can mediate insulin sensitivity, adipose tissue lipolysis, inflammation and adipokine production. It can also promote liver gluconeogenesis and glycogenolysis and suppress glucose utilization and glycogen stockpiles. High-fat diet and IR affect the H_2_S setup in the hepatic and adipose tissue which highly depends on diet composition [68]. In addition, these enzymes are expressed in insulin-secreting pancreatic β cells, and they can suppress insulin secretion by activating ATP-sensitive K+ channels and thus have pro- or antiapoptotic effects on β cells. Research shows that excessive pancreatic H_2_S may be conducive to T2DM development [69]. The principal generators for H_2_S production are represented by sulfate-reducing gut bacteria such as *Desulfobacter*, *Desulfobulbus*, *Desulfovibrio* and *Desulfotomaculum*. H_2_S also results from cysteine catalyzation by certain anaerobic bacterial strains (*Salmonella enterica*, *Escherichia coli*, *Clostridia* and *Enterobacter aerogene*), or from different tissues via cystathionine gamma-lyase (CSE), cystathionine beta-synthase (CBS) and 3-mercaptopyruvate sulfurtransferase (3-MST) [70]. An in vitro study showed that surplus of H_2_S levels inhibited pancreatic cells function and therefore has a pro-diabetic role [71]. In contrast with these results, via downregulation of thioredoxin-interacting protein expression, H_2_S protected the mouse pancreatic cells exposed to raised glucose or fatty acids, implying the antidiabetic role of H_2_S [72]. The potential role of H_2_S in T2DM development is far from being established, and, to elucidate the exact involvement of this GM generated-product, further research is desired.

### 4.6. Immune System in GM-T2DM

Among the mechanism involved in GM dysbiosis and T2DM is an imbalance in the immune system. GM and its metabolites maintain the balance and function of intestinal T helper 17/regulatory T cell (Th17/Treg) and gut-associated lymphoid tissues (GALT) such as Peyer’s patches, mesenteric lymph nodes and isolated lymphoid follicles [73]. GM is essential in helping the immune system to differentiate self from non-self (invaders), and it promotes innate hematolymphoid cells (ILC1, -2 and -3), natural killer (NK) cells, cytotoxic and noncytotoxic cells and helper lymphoid cells synthesis. GM metabolites such as tryptophan and α-galactosylceramide enhance immune organelles to produce IL-17, IL-12 and immunoglobulin A (IgA), which help prevent infiltration of certain microorganism into the bloodstream [74,75]. The Innate Lymphoid Cells (ILCs) are vital components of innate immunity that produce both regulatory and pro-inflammatory cytokines to promote tissue repair, immunity and inflammation. Research shows that T2DM subjects have increased circulating and tissue adipose levels of ILCs1 and its high levels are correlated with increased risk of diabetes onset [76,77], whereas ILC2s may improve glucose homeostasis, resulting in both protection against IR onset and amelioration of established IR [78].

Intestinal Th17 cells are able to modulate glucose homeostasis, adipogenesis, control immune tolerance and participate in IR development via a reduction of intestinal RORγt+ and IL-17-producing CD4+ [73,75]. Additionally, various studies demonstrated how GM and its products can regulate the harmony of intestinal Th1/Th2 cells functions. In this matter, a commensal A4 bacteria part of the *Lachnospiraceae* family by induction of dendritic cell TGF-β production can produce a CBir1 antigen that can inhibit the intestinal Th2-cell responses [79]. In addition, *Bacteroides fragilis* produces a polysaccharide A, which can enhance the synthesis of proinflammatory cytokines such as p40 and IL-12 that promote Th1 activation [80]. IL-12 owns a pivotal role as an immunoregulatory factor in T2DM and its complications. Via the signal transducer and activator of transcription 4 (STAT4) signaling pathway, IL-12 attaches to its receptors on the pancreatic β-cells, triggering proinflammatory cytokines and inducing cell apoptosis [81]. The disruption of IL-12 promotes angiogenesis in obese type 2 diabetic rodents by an endothelial nitric oxide synthase/Akt/vascular endothelial growth factor receptor 2/oxidative stress-inflammation-dependent process [78].

**Figure 1:**
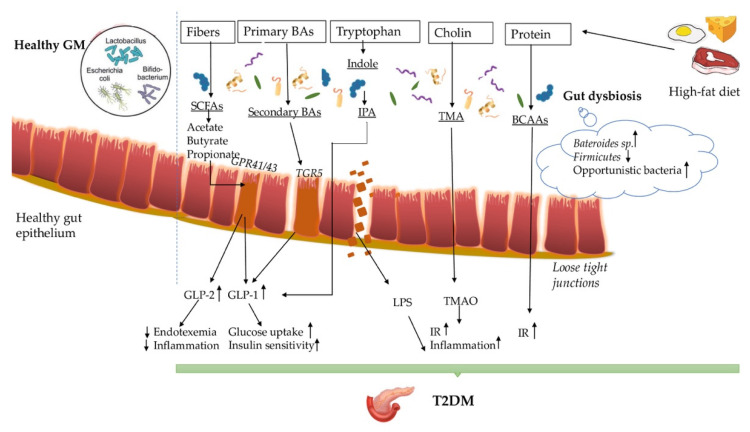
The complex association between gut dysbiosis and T2DM. Short fatty acids (SCFAs); G-protein-coupled receptors 41/43 (GPR41/43); glucagonlike peptide-1 (GLP-1); glucagonlike peptide-2 (GLP-2); G protein-coupled bile acid receptor 1 (TGR5); Indole-3-propionic acid (IPA); Insulin resistance (IR); Trimethylamine (TMA); Trimethylamine N-oxide (TMAO); Branched-chain amino acids (BCAAs)**.**

The gut–diabetic axis and the underlying immunopathogenesis are very intriguing and involve many various commensal intestinal bacteria that carry homeostatic and metabolic functions, which in dysbiosis conditions through different products and metabolites can endorse and maintain diabetes. GM help with the metabolizations from dietary fibers of SCFAs (butyrate, acetate and propionate) that can increase the glucose uptake and insulin sensitivity and/or decrease inflammation and endotoxemia. GM contributes to the formation of secondary BAs from primary BAs and to the production of IPA from tryptophan, both of which can activate the secretion of GLP-1. Moreover, dietary choline and proteins are metabolized by GM into TMAO and branched-chain amino acids, which in high levels can instigate inflammation and IR. Lastly, the GM has a key role in maintaining and modulating the balance of the innate immune system responses in T2DM (Figure 1).

## 5. Gut Microbiota and Diabetic Retinopathy

Diabetic retinopathy (DR) affects up to 60% of T2DM patients, with a higher prevalence in T1DM subjects and is the main instigator of blindness including severe retinopathy and diabetic macular edema. DR is defined by microaneurysm, raised vascular permeability, intraretinal hemorrhages, exudates, dysregulations in the venous caliber, intraretinal microvascular abnormalities and neovascularization. In addition, the neurosensory component of the retina is impaired in diabetes [82,83]. The ocular surface is a complex ecosystem that is influenced daily by numerous environmental factors. Complex interplays among microvascular, neurodegenerative, immunological, genetic/epigenetic and inflammation-related factors are identified in DR development [84]. For many years, the precise ocular microbiota has been debated by many ophthalmologists; however, no consensus has been reached so far. Studies reported that the ocular surface holds around 12 genera of bacteria and the principal microbiome constituents are represented by *Corynebacterium* and coagulase-negative *Staphylococci, Acinetobacte* spp. as well as “pathogenic” bacteria such as *Pseudomonas* [85,86].

The ocular microbiome has important functions in the conjunctiva and cornea, especially in mediating eye immunity and in pathogens prevention [87]. Studies showed that diabetic patients with complications have higher bacterial conjunctival flora compared to T2DM-patients without [88]. The pathogenesis and development of DR are extremely complicated. Multiple tangled mechanisms that persuade cell injury and cellular adaptive changes in the retina are involved. Functional and structural changes in microvascular and neuroglial components are identified, however, there are fundamental mechanisms that are still elusive [88]. Beli et al. [89] described the first conclusively report of the association between the GM and DR. Rodents on the ad libitum diet exerted manifestations of diabetic retinopathy such as infiltration of inflammatory cells and acellular capillaries, whereas intermittent fasting mice had retinal histology similar to non-diabetic controls. Intermittent fasting rodents also exhibited an increased ratio of *Firmicutes* to *Bacteroidetes* on 16S rRNA assays, and modifications of bacterial metabolites increased levels of taurochenodeoxycholate (TUDCA), a bile acid metabolite with known anti-inflammatory effects. TUDCA enters the circulation and can activate GPBAR1, also known as TGR5, the receptor for TUDCA in the retina. The results may suggest that intermittent fasting may protect against DR by increasing the levels of TUDCA and also, TGR5 could represent a new therapeutic target for the diabetic retina [90]. The mechanisms for the association between T2DM and gut dysbiosis were mention above, however, these alterations have not been directly linked to DR just the intermediary products. Several experimental and research studies highlight how gut dysbiosis is involved in T2DM pathogenesis; however, the link between gut commensals and DR is far from being elucidated [91,92,93].

## 6. Gut Microbiota and Diabetic Neuropathy

T2DM induces neurodegeneration; the neuronal function is disrupted, leading to a pathological glucose detection that aggravates the evolution of the disease. Diabetic neuropathy is the result of demyelination, axonal atrophies and inflammation. It is mostly represented by distal polyneuropathy (DPN) that occurs in 30–50% of diabetic patients after long-term exposure to hyperglycemia. DPN manifests by decreasing sensitivity and motor function in limb extremities, and it can be associated with impaired kinesthesia, mobility dysfunction, numb sensation or even pain [94,95,96]. Although this is the most common chronic nerve degradation due to T2DM, in the last decades, we have tried to observe how T2DM determines alterations of the nervous system, especially the autonomic one, and influences the gut–brain axis activity. T2DM-induced neuropathy can also manifest at a gastrointestinal (GI) level. It affects different parts of the GI tract and it mostly manifests by constipation or diarrhea, abdominal pain, nausea and sometimes vomiting [97]. One of the causes of these pathological changes is the shift observed in the GM due to DM [98].

The main elements of the nervous system that are able to recognize microbiota components and molecules are represented by the enteric nervous system (ENS) and the vagus nerve (VN). ENS is formed by neurons and glial cells that are found in the myenteric and submucosal parts of the GI tract. Afferent (sensory neurons) and efferent (sympathetic and parasympathetic pathways) fibers keep ENS connected to the central nervous system [99]. There are also the interstitial cells of Cajal that generate the electric activity of the smooth muscles, resulting in the peristaltic movement [100]. In addition, the entero-endocrine cells play a major role in the microbiota influence of the neuronal activities [101]. The interaction between the microbiota and the host is still not fully understood, but there are a few studied mechanisms through which the microbiota produces different molecules such as SCFAs, neuro-hormones or mimetics (GABA and Caseinolytic protease B) and imidazole propionate [44,102]. It was also shown that the GM has a low-grade inflammation effect through its own components such as LPS through TLR4 and CD14 or peptidoglycans. The latter has an important role in the variations of insulin sensitivity by modulating intestinal colonization through NOD2 [103]. Some bacterial ribonucleic acid (RNA) and deoxyribonucleic acid (DNA) fragments also bind to TLR [104]. Through these complex mechanisms, the GM has a strong influence not only on the gastrointestinal transit but also on neuronal homeostasis (Table 1). The ENS structural and functional integrity seems to depend on TLR4 and nuclear factor–κB activation using microbiota components such as LPS. TLR2 activation by different microbial products regulates the intestinal neuromuscular function. In addition, the role of TLR9 in the neuromuscular junction development is considered a key element in neuronal activity [105,106,107]. Some studies have shown that mice that were administrated antibiotics, as well as germ-free (GF) mice, had an immature ENS with weak responses to different stimuli. Not only their ENS, but also their VN, did not respond to neuro-hormones. The constant information transfer between the nervous system and the microbiota needs to be maintained for a healthy gut-brain signaling. GLP-1 is used in T2DM patients to control the glycemic level. In animal models, gastric emptying and insulin secretion are strictly related to the GLP-1 receptor and the functional synthesis of neuronal nitric oxide (NO). Their normal activity is possible within a eubiotic gut environment [108,109,110].

The importance of SCFAs is taken into consideration in the latest research. In a 2016 study, the authors supplemented the diet of GF mice with SCFAs, and it induced chromatin changes affecting the host epigenome similar to those associated with colonization [111]. Focusing on epigenetics, Wahba and colleagues studied the role of methyl CpG binding protein (MeCP2) in the development of the ENS. The mutation of the MeCP2 gene leads to a disruption of the neuronal communication known as Rett syndrome (RTT). In addition, the fact that MeCP2 is expressed throughout the ENS explains the dysmotility associated with RTT. An important imbalance of the NO synthesized at neuronal level was also observed in the MeCP2 mutation that can also be a cause for the dysregulation of the GI transit [112,113]. GLP-1 action is strongly influenced by NO, which is why MeCP2 could have a role in the physiology of glucose metabolism [110].

The myenteric neurons play an essential role in the well-functioning of GI motility by signaling the smooth muscles and coordinating the movements at different levels of the GI tract [114]. Dysmotility is due to apoptosis or necrosis of the myenteric inhibitory motor neurons. The damage causes disorganization of the neuropil, loss of cytoskeletal filaments and axonal swelling [115]. T2DM is usually associated with a western high-fat diet, which is why most studies use animal models that are on a high-fat diet (HFD). ENS injuries induced by HFD in mice and rats were similar to those in diabetic humans. In a similar experiment, it was observed that high palmitic acid exposure is a contributing factor in diabetic neuropathy, causing gastrointestinal dysregulation [116,117,118,119]. Until recently, most ENS injuries associated with T2DM or HFD were thought to be caused by oxidative stress related to inflammation and hyperglycemia, but, in the last years, there has been growing interest in the role of the GM in the pathophysiology of T2DM. The two main molecules that seem to have a fundamental role in T2DM development associated with GI neuropathy and dysmotility are LPS and a saturated fatty acid called palmitate. Some studies actually suggest that neuropathy occurs before any T2DM symptoms [120,121,122]. The association between dysbiosis, nerve cell damage and dysmotility is not yet fully understood, but the GM definitely has an important role in maintaining a functional ENS [123,124]. In a recent study, Nyavor and colleagues observed how T2DM induces gastrointestinal disorders by affecting the enteric nerves. HFD-fed mice had a decrease in the inhibitory neuromuscular transmission and lost myenteric inhibitory motor neurons. The HFD also induced dysbiosis with an increase in the number of *Allobaculum*, *Lactobacillus* and *Bifidobacterium*. All these changes correlated with neuropathy and intestinal dysmotility [125].

**Table 1:** Gut microbiota and neuropathy implications. Glucagonlike peptide-1 (GLP-1); nitric oxide (NO); enteric nervous system (ENS); toll-like receptor (TLR); lipopolysaccharide (LPS); germ free (GF); short-chain fatty acids (SCFAs); methyl CpG binding protein (MeCP2); neuronal nitric oxide synthase (nNOS); gastrointestinal (GI); high fat diet (HFD); ribonucleic acid (RNA); deoxyribonucleic acid (DNA).

Main Focus	Species	Salient Findings	Country	Year and Reference
therapy	mice	GLP-1based therapy for functional insulin secretion requires an eubiotic intestinal microbiota environment	France	2017
				[110]
neuropathy	mice	The ENS structural and functional integrity depends on TLR4 and nuclear factor–κB activation using microbiota components such as LPS	USA	2012
				[105]
neuropathy	mice	TLR2 activation by different microbial products regulates the intestinal neuromuscular function	Italy	2015
				[106]
neuropathy	mice	TLR9 in the neuromuscular junction development is considered a key element in neuronal activity	USA	2016
				[107]
neuropathy	mice	Normal functioning of intestinal neurons requires commensal intestinal microbiota	Canada	2013
				[108]
neuropathy	mice	Functional gut-brain signaling requires an intact microbiome	Canada	2015
				[109]
diet/therapy	mice	Diet supplementation with SCFAs in GF mice induced chromatin changes affecting the host epigenome similar to those associated with colonization	USA	2016
				[111]
neuropathy	humans + murine	Mutation of the MeCP2 gene leads to a disruption in the neuronal communication that explains the intestinal dysmotility in Rett syndrome	Canada	2015
				[112]
neuropathy	mice	An important imbalance of the nitric oxide synthesized at neuronal level was observed in the MeCP2 mutation that can be a cause for the dysregulation of the GI transit	Canada	2016
				[113]
diet/diabetic neuropathy	mice	High palmitic acid exposure is a contributing factor in diabetic neuropathy causing gastrointestinal dysregulation	Sweden	2013
				[116]
diet/neuropathy	mice	HFD-fed mice had a decrease in the inhibitory neuromuscular transmission and lost myenteric inhibitory motor neurons correlated with intestinal dysbiosis	USA	2020
				[125]
neuropathy	murine	The enteric neural network can be activated directly by bacterial RNA and DNA fragments that bind to TLR	Italy	2009
				[104]
diet/neuropathy	mice	HFD-induced dysmotility is due to apoptosis or necrosis of the myenteric inhibitory motor neurons	USA	2013
				[115]
diet/diabetic neuropathy	mice	Type 2 diabetes and HFD influence the ENS; diabetic dysmotility is caused by nerve damage	USA	2015
				[117]
diabetic neuropathy	humans	Oxidative stress causes loss of enteric neurons that may be inducing diabetic dysmotility; antioxidants are a possible therapeutic option in diabetic motility disorders	USA	2011
				[118]
diet/neuropathy	mice	Western diet induces neurodegeneration and dysmotility through TLR4 activation, even without overt endotoxemia or hyperglycemia	USA	2017
				[122]
neuropathy	mice	Early exposure to intestinal microbiota is necessary for physiological development of the ENS	Canada	2014
				[126]

## 7. Gut Microbiota and Diabetic Nephropathy

Diabetic nephropathy (DN) is a chronic condition that occurs in approximately 40% of diabetic individuals. It is mostly characterized by reduced glomerular filtration rate and increased albuminuria. Both T2DM and chronic kidney disease (CKD) have as a common pathophysiological mechanism a chronic inflammatory state [126]. It is considered that the increased intrarenal renin-angiotensin system (RAS) activation is one of the main causes of DN. Higher renal sensitivity to angiotensin II leads not only to systemic and glomerular hypertension but also to phenotypic alteration of the podocytes and glomerular endothelial cells with inflammatory and profibrotic factors secretion that accelerate the DN progression [127,128].

In individuals with a predisposition for CKD such as those with T2DM, intestinal dysbiosis represents a susceptibility factor for the development of nephropathy [129]. In addition, progressive kidney disease determines alterations of the GM [130]. Activation of the GLP-1/GLP-1 receptor complex attenuates proximal tubular reabsorption and growth, ameliorating the early manifestation of DN [131]. SCFAs bind to GPCRs, as well as to an olfactory receptor (Olfr78). The best known and studied GPRs are GPS43, GPR41 and GPR109. The smooth muscles that can be found at renal arteries level express mostly GPR41 and Olfr78. Intrarenal RAS can be activated by the propionic acid resulted from the intestinal flora that binds to the Olfr68. This leads to increasing the glomerular and systemic pressure that can be a contributing factor in developing DN [132].

Recently, some studies have tried to comprehend the implications of phenyl sulfate (PS) in DN. PS is considered a uremic toxin derived from the GM. It is correlated with early kidney damage in diabetic patients, making it a valuable potential marker to identify diabetic individuals that are at risk of developing DN. Furthermore, its involvement in different molecular mechanisms that lead to podocyte injury makes targeting its intestinal production a possible pharmaceutical option [133].

A study conducted in 2019 on rats explored the close pathophysiological mechanisms that connect early DN with the intestinal microbiota alterations. DN was induced by intraperitoneally injecting male rats with streptozotocin. After that, for eight weeks, they were orally administrated antibiotics and their kidney tissue along with blood, urine and fecal samples were analyzed. Rats with induced DN had an increase in plasma acetate levels with abnormal intestinal microflora. At the renal level, the glomerular basement membrane was thickened, the podocyte foot process was obliterated and mesangial matrix hypertrophy and an increase in the angiotensin II type 1 receptor were observed. In addition, the levels of angiotensin-converting enzyme and angiotensin II were increased, along with proteinuria. Antibiotic administration eliminated most of the gut microflora, decreasing plasma acetate levels, inhibiting the renal RAS and attenuating kidney damage. The disturbed microbiota increased the production of acetate in DN rats, causing the early kidney injuries of DN by activating the renal RAS [134].

In another study, Hu and colleagues focused on how GM dysbiosis leads to the disruption of cholesterol homeostasis that promotes DN. Tubulointerstitial injury and albuminuria were attenuated in diabetic rats treated with antibiotics. In addition, they had a decrease in the acetate serum levels that correlated with renal cholesterol accumulation. Fecal microbiota transplantation from a healthy donor group had a similar effect on cholesterol metabolism and renal impairment of diabetic rats. An in vitro experiment on human kidney 2 cells that were stimulated with sodium acetate showed that acetate through GPR43 activation promoted cholesterol homeostasis [135].

A Chinese study used mice with DN induced by streptozotocin to analyze the microbiota influence on renal function. The mice were split into two groups according to the proteinuria level. There were mice with severe proteinuria (SP) and others with mild proteinuria (MP). There was different microbiota composition in the two groups. *Firmicutes* phylum had a reduced abundance in the SP group, meanwhile, in the MP group, an increase was observed. *Allobaculum* was significantly enriched in the SP group compared to the MP one. This led to body weight gain and an increase in glucose levels, accelerating the evolution of DN. *Anaerosporobacter* is another genus that was abundant in the SP group [136]. It is correlated with an increase of TMAO in the blood that leads to the formation of vascular plaques and intestinal mucosa damaging. Given the vascular structure of the kidney, the renal function and structure are very vulnerable to TMAO actions. Intestinal mucosa destruction promotes TMAO transport into the blood flow that causes oxidative stress, adding even more damage to the kidney [137,138]. All these harmful effects are increased by LPS production. In the MP group, *Blautia* was the dominant microbiota constituent. This may lead to producing propionic and butyric acids that reduce proteinuria in DN mice. SCFAs have a protective effect on the renal function of patients with CKD. Butyrate also improves the intestinal barrier, reducing LPS and TMAO influx into the blood, attenuating DN [139].

Diet has a crucial role in maintaining the intestinal microbiota in balance. Fibers are the source of bacterial fermentation, but patients with CKD have a reportedly low dietary fiber intake. This may lead to an increase in the intestinal transit time, which promotes carbohydrates fermentation in the small intestine, making them less available for the colonic bacteria. Protein absorption in the proximal segments of the intestine is impaired in CKD individuals, increasing the protein availability for bacteria in the colon [139,140,141]. Changes in GM were observed in the early stages of CKD. In the small bowel, there was an overgrowth of aerobic and anaerobic bacteria. At the colonic level, an increase in the counts of *Actinobacteria*, *Proteoteobateria* and *Firmicutes* was documented. It was represented mostly by species that have urease, uricase, p-cresol and indole-forming enzymes [142,143]. The role of GM in the development of CKD has been researched in recent years (Table 2).

## 8. Therapy and Future Perspectives

Current knowledge and studies indicate that targeting GM could provide a new therapeutic strategy in battling T2DM and its complications along with the already known treatment. Starting from the concept that GM may promote IR and glucose metabolism alterations, readjusting the microbiota or its direct modulation could benefit this chronic disorder. However, it is difficult to assess a straight therapeutic option to manage T2DM as many patients get to use antibiotics which negatively interfere with GM. Therefore, scientists directed their attention to the valuable role of prebiotics and probiotics. They replenish depleted taxa by bringing more beneficial bacteria that can improve gut metabolic functions (i.e., production of anti-microbial lactic acid, improvement of intestinal barrier function, inhibition of α-glucosidase activity, immune modulation, SCFAs production and modulation of bile acid metabolism), proving they can be effective in T2DM management [144].

Experimental studies and many randomized controlled trials showed that *L. rhamnosus*, *L. gasseri* and *L. casei* [145], as well as *Lactobacillus acidophilus*, *Streptococcus thermophilus*, *L. bulgaricus* and/or *Bifidobacterium lactis*, administered for 6–12 weeks exert antidiabetic effects [146]. In addition, the use of *Bifidobacterium animalis*, *B. longum* and *B. breve* alleviated glucose intolerance [147], while *A. muciniphila* and *F. prausnitzii*, which are negatively associated with hyperglycemia and overweight, may be potential candidates for next-generation probiotics [148]. As the already known strategies for diabetic retinopathy therapies mainly include late-stage disease, new therapeutic approaches are needed. The authors showed that extended (12 weeks) administration of a specific probiotic, *Lactobacillus rhamnosus*, leads to decreased glucose plasma levels and intraocular pressure compared to control in chronic disease models of type 1 diabetes [149]. In addition, after flora is transferred from diabetic mice to colonize GF ones, the GLP-1-related glucose metabolism regulation is alerted. Diabetic neuropathy induced by gut dysbiosis alters the efficacy of antidiabetic treatments [150]. This evidence shows the beneficial effects of probiotics and prebiotics on diabetic individuals that could also help in the management of its related complications. Further larger trials are needed before laying out this strategy.

One forward method that directly influences GM composition and may ameliorate insulin sensitivity and therefore alleviate diabetes, is fecal microbiota transplant (FMT). After studying this approach, the authors proved in well-controlled randomized clinical trials that FMT ameliorates insulin sensitivity in healthy subjects. These results also show that the beneficial effects of FMT are transient and highly dependent on host individualized response [151]. Transplantation of *F. prausnitzii* showed beneficial results on GM pattern, it can restore the intestinal barrier structure and function through the regulation of the tight junction pathway, and it may be used as a potential therapeutic approach against inflammation and diabetes [152,153].

Given the fact that the intravenous form of Metformin, a GLP-1 agonist used as an antidiabetic treatment, does not control hyperglycemia but only the oral form does, this implies that the intestinal tract is an important site of Metformin action [154]. In addition, this drug alters the GM by making it look similar to that of healthier subjects [155,156]. Another approach evaluated the association between Metformin and *Radix Scutellariae*, a traditional oriental medicine. It reduced the harmful bacteria levels, hence it improved bile acid homeostasis, lipid metabolism and obesity-induced IR [157]. The combination of Oligofructose and Metformin improves glycemic control and promotes weight reduction, which sounds promising as a novel strategy for reducing side effects induced by the metabolic syndrome [158].

Some traditional Chinese medicine, used for years to treat T2DM, seem to ameliorate inflammation, hyperglycemia and gut dysbiosis in T2DM rodents by decreasing levels of pathogenic bacteria such as *Staphylococcus*, *Aerococcus* and *Corynebacterium*; increasing the number of anti-inflammatory bacteria such as *Akkermansia* and *Blautia*; and reducing glycolysis/gluconeogenesis and nucleotide metabolism [159]. An experimental study on rodents showed that administration of a purified anthraquinone-glycoside resulted from Rhubarb ameliorated gut dysbiosis, raising the probiotic Lactobacillus levels and SCFA-producing bacteria, and it has hypoglycemic effects by mediating gut microbiota and by activating the GLP-1/cAMP pathway to ameliorate IR [160]. In a 2020 study performed on rodents with diabetic nephropathy, Cordyceps cicadae polysaccharides (CCP) modulated the composition of the GM and improved glucose tolerance along with IR. Furthermore, it suppressed inflammation by reducing cytokine levels that were induced by LPS. In addition, tubulointerstitial fibrosis was decreased by reducing the transforming growth factor-beta 1 (TGF-β1) [161]. Alternative therapies that influence GM composition are emerging. In a recent study, Meng and colleagues proved that Bekhogainsam decoction (BHID) affected the intestinal flora of DN mice, acting through phosphatidylinositol-3-kinase/protein kinase B and other protein targets related to MAPK. It also prevented kidney dysfunction, making it a great support in the clinical treatment of diabetic patients [162]. Jowiseungki decoction (JSD) is another possible option for ameliorating DN symptoms. It decreases renal inflammation and fibrosis by regulating nuclear factor-κB/α-SMA and protein kinase C-alpha/phosphatidylinositol-3-kinase/protein kinase B signaling pathways. In addition, JSD has an impact on modifying the intestinal bacterial composition [163]. In another study conducted by Zhao and his colleagues, they concluded that Tangshen Formula, a Chinese herbal medicine, modulates the GM and decreases renal inflammation. It reduces LPS and indoxyl sulfate levels, contributing to intestinal toxins elimination in DN patients [164].

Quercetin is a flavonoid with powerful antioxidant properties that was recently studied for its neuroprotective function and prebiotic capacity. It was tested on rats with diabetic peripheral neuropathy induced by streptozotocin administration. The results show that quercetin exerts a neuroprotective effect by ameliorating the damage caused by oxidative stress at an axonal level. It also has an impact on intestinal dysbiosis by modulating the GM associated with reactive oxygen species production and diabetic peripheral neuropathy phenotypes [165]. Berberine is a quaternary ammonium salt extracted from different plants that is used in China for its anti-inflammatory and antioxidant properties. It was shown that it has a hypoglycemic effect on T2DM patients by reducing glucose intestinal absorption and improving insulin sensitivity. In addition, it increases insulin secretion and influences the glucidic metabolism at a hepatic level by modulating peroxisome proliferator-activated receptors expression. It was proven to induce a hypoglycemic effect by balancing the GM, and its antioxidant effects combat diabetic complications such as diabetic neuropathy and diabetic kidney disease [166,167].

In a recent study, the authors tested dietary fibers and their effects on DN. Streptozotocin-induced diabetic mice that were on a high-fiber diet had a significant chance of not developing DN compared to those that were under normal chow or zero-fiber diet. Fibers promoted the growth of *Bifidobacterium* and *Prevotella*, increasing not just fecal but also systemic concentrations of SCFAs. They also reduced inflammation by suppressing the encoding genes for cytokines, chemokines and proteins that promote fibrosis at a renal level in diabetic conditions. The diabetic mice treated with SCFAs were also protected from developing nephropathy, but only in the presence of GPR43 or GPR109A gene [168,169].

New therapies for diabetic-associated neuropathy, nephropathy and retinopathy are emerging. An integrative medicine approach may improve the patient’s symptomatology. Until further research is made, a healthy lifestyle with regular exercise and a balanced diet is one of the main recommendations. A gut-microbiota-based T2DM parameter might help with T2DM diagnosis and monitoring. Administration of pre- and probiotics as well as antibiotic therapy should be individually tailored to prevent or even treat chronic diseases such as diabetes.

## 9. Conclusions

The cumulated results underline the role of diabetes in vascular impairment with subsequently macrovascular and microvascular complications. In the long run, hyperglycemia damages certain organs and systems, especially the heart, kidneys and eyes. Recent studies show that abnormal plasma glucose levels can be modulated by GM, thus there is a high chance that GM plays a particularly special role in mediating diabetes. In the past years, many gut microbiota transplantation experiments, not just on animal models but also on humans, have shown that dysbiosis can be both the cause and the consequence of T2DM and its vascular complications.

As seen, diabetic subjects have decreased butyrate-producing bacteria levels such as *Akkermansia* and *Bifidobacterium* and of *Lactobacillus* spp., *Bifidobacterium* spp. and *Verrucomicrobia* phyla, microorganisms that have anti-inflammatory gut effects. Moreover, most of the studies pointed out that T2DM patients have increased Gram-negative bacteria such as *Bacteroides* that can alter the intestinal mucus and glycocalyx barrier enabling the pathogen to enter the circulation and that decreased *Akkermansia muciniphila* could be used as a biomarker for the early diagnosis of diabetes. These results suggest that the growth of beneficial bacteria in patients with healthy diets, or by addition through pre- and probiotics, or even by FMT, could protect against T2DM development. They can also represent a new addition to the pharmacological diabetic treatment. In addition, abnormal production of GM metabolites, such as SCFAs, TMAO, LPS, H_2_S and aromatic amino acids, seems to contribute to T2DM pathogenesis through metabolic and immunologic pathways. For example, as described above, manipulation of BAs via adjustment of the GM could help glycemic control, thus BA sequestrants (i.e., colesevelam and obeticholic acid) have been introduced recently as therapeutic options in patients with inadequately controlled diabetes and individuals with diabetic retinopathy. There was an increase in the interest of the scientific community for alternative therapies including traditional medicine, antioxidants and different compounds such as oligofructose. Used alone or in association with Metformin, they proved their efficacy in preventing DM development and its microvascular complications, which opens the way for new ideas and hopes for future studies.

However, there is still a paucity of information available that can help develop personalized T2DM therapy based on microbial composition and/or metabolites. The alluring and intriguing use of the gut microbiome as a target for the prevention, diagnosis and treatment of T2DM needs further research. In the past few years, the focus on the GM–diet–host metabolism triangle seems to lead to a novel GM-based T2DM parameter/index and a detection platform (XenoScan) that together would act as an additional tool/predictive biomarker that establishes the risk of diabetes onset and helps us find new therapy targets among the gut microbial ecosystem.

## Figures and Tables

**Table 2:** Gut microbiota and diabetic nephropathy. Gut microbiota (GM); G protein-coupled receptor (GPR); diabetic nephropathy (DN); diabetes mellitus (DM); renin–angiotensin system (RAS); short-chain fatty acids (SCFAs); glucagonlike peptide (GLP); lipopolysaccharide (LPS).

Main Focus	Salient Findings	Country	Year and Reference
physiopathology	The disturbed microbiota increased the production of acetate in DM rats, causing early kidney injuries of DN by activating renal RAS	China	2020
			[134]
physiopathology	Tubulointerstitial injury present in DN is induced by high acetate levels produced by the GM	China	2020
			[135]
physiopathology	Specific bacteria from the intestinal microbiota influences the renal function in mice with diabetic kidney disease	China	2020
			[136]
physiopathology	Activation of the GLP-1/GLP-1 receptor complex attenuates proximal tubular reabsorption and growth, ameliorating early manifestation of DN	USA and Ireland	2014
			[131]
physiopathology/therapy	Phenyl is correlated with early kidney damage in diabetic patients, making it a valuable potential marker to identify diabetic individuals that are at risk of developing DN; its involvement in different molecular mechanisms that lead to podocyte injury makes targeting its intestinal production a possible pharmaceutical option	Italy	2020
			[133]

## Abbreviations

T2DM	type 2 diabetes mellitus
IR	insulin resistance
GM	gut microbiota
SCFAs	short chain fatty acids
TMAO	trimethylamine N-oxide
GLP-1	glucagonlike peptide-1
LPS	lipopolyscaccharide
MR	Mendelian randomization
PG	peptidoglycan
TLR-4	toll-like receptor-4
GPR	G-protein-coupled receptors
BAs	bile acids
CDCA	chenodeoxycholic acid
CA	cholic acid
DCA	deoxycholic acid
LCA	lithocholic acid
FXR	farnesoid X receptor
TMA	trimethylamine
FMO3	flavin monooxygenase 3
TMAO	trimethylamine oxide
VLDL	very-low-density lipoproteins
HOMA-IR	homeostasis model assessment of insulin resistance
BCAAs	Branched chain amino acids
IPA	3-Indolepropionic acid
H_2_S	Hydrogen Sulfide
Th17/Treg	T helper 17/regulatory T cell
GALT	gut-associated lymphoid tissues
NK	natural killer
ILCs	The Innate Lymphoid Cells
STAT4	signal transducer and activator of transcription 4
DR	Diabetic retinopathy
TUDCA	taurochenodeoxycholate
DPN	distal polyneuropathy
DM	diabetes mellitus
GI	gastrointestinal
ENS	enteric nervous system
VN	vagus nerve
RNA	ribonucleic acid
DANN	deoxyribonucleic acid
GF	germ-free
MeCP2	methyl CpG binding protein
RTT	Rett syndrome
HFD	high fat diet
NO	nitric oxide
nNOS	neuronal nitric oxide synthase
DKD	diabetic kidney disease
CKD	chronic kidney disease
RAS	renin-angiotensin system
DN	diabetic nephropathy
Olfr78	olfactory receptor Olfr78
PS	phenyl sulfate
SP	severe proteinuria
MP	mild proteinuria
CCP	cordyceps cicadae polysaccharides
TGF-β1	transforming growth factor-beta 1
FMT	fecal microbiota transplantation
BHID	Bekhogainsam decoction
JSD	Jowiseungki decoction
MAPK	mitogen-activated protein kinase
